# Supplementary material for: Predicting visual function by interpreting a neuronal wiring diagram
Source: Nature. 2024 Oct 2;634(8032):113–23. doi: 10.1038/s41586-024-07953-5 (PMC11446822; doi:10.1038/s41586-024-07953-5)
Supplement: Supplementary file 6 — CRF and ERF predictions for individual TmY4 and TmY9 cells. Analogous to Supplementary Data 3, but for TmY target types. Shown are the top four monosynaptic pathways, the strongest pathway passing through each of the top ten intermediary types (ranking from Extended Data Fig. 7), and the trisynaptic pathway Tm1–TmY–Dm3–TmY (see the section entitled Prediction of spatial normalization). [file 41586_2024_7953_MOESM6_ESM.zip › DataS4/TmY9q/720575940626145555.pdf]

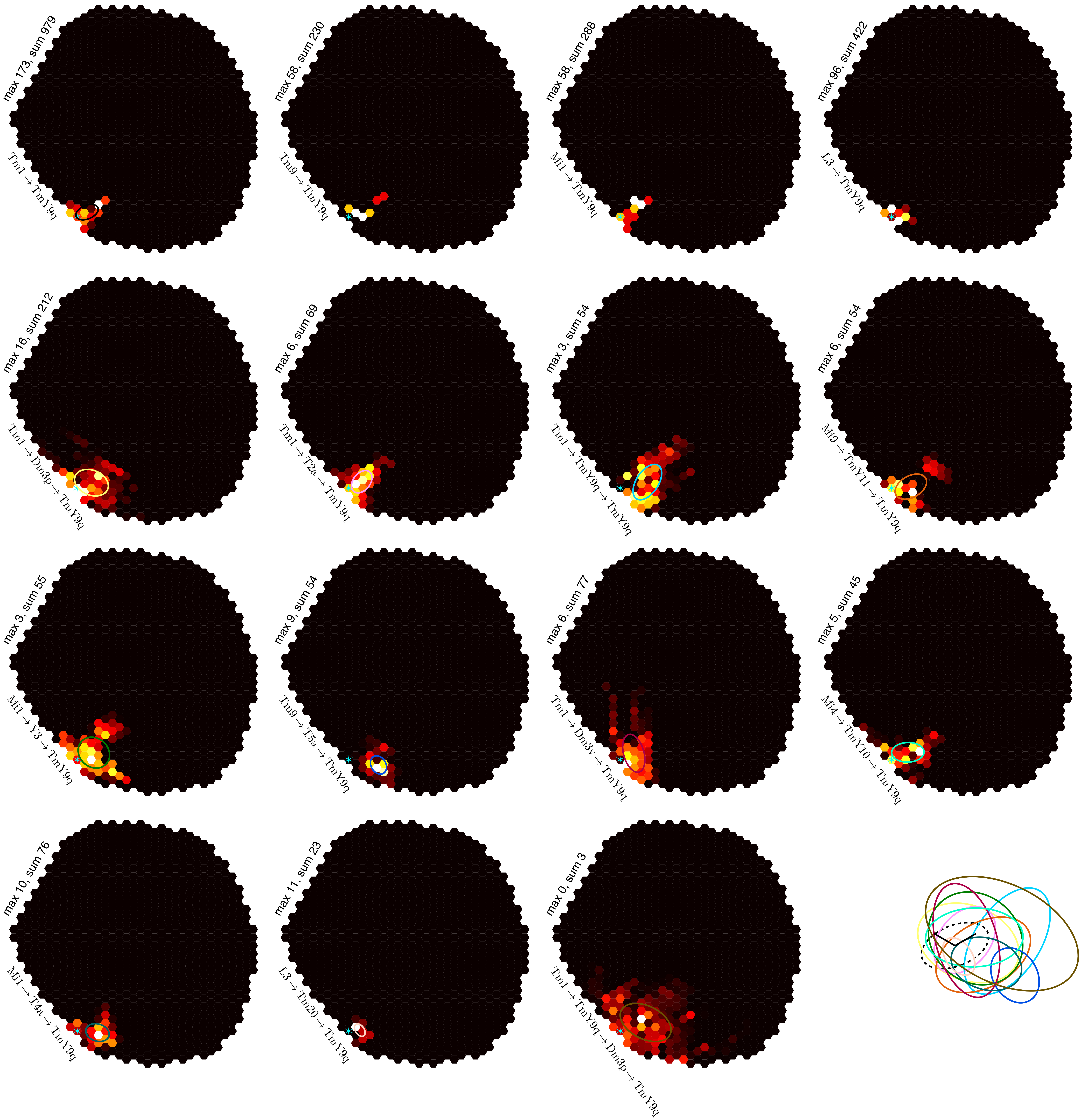

max 173, sum 979
Tm1 → TmY9q
max 58, sum 230
Tm9 → TmY9q
max 58, sum 288
Mi1 → TmY9q
max 96, sum 422
L3 → TmY9q
max 16, sum 212
Tm1 → Dm3p → TmY9q
max 6, sum 69
Tm1 → T2a → TmY9q
max 3, sum 54
Tm1 → TmY9q → TmY9q
max 6, sum 54
Mi9 → TmY11 → TmY9q
max 3, sum 55
Mi1 → Y3 → TmY9q
max 9, sum 54
Tm9 → T5a → TmY9q
max 6, sum 77
Tm1 → Dm3v → TmY9q
max 5, sum 45
Mi4 → TmY10 → TmY9q
max 10, sum 76
Mi1 → T4a → TmY9q
max 11, sum 23
L3 → Tm20 → TmY9q
max 0, sum 3
Tm1 → TmY9q → Dm3p → TmY9q
